# Energy-Efficient Microwave Heating: Governing Role of the Thermal Profile in Heating Liquid

**DOI:** 10.1021/acsomega.6c00849

**Published:** 2026-05-26

**Authors:** Song Jia, Kama Huang, Song Wang, Haoyu Wang

**Affiliations:** † School of Electronics and Information Engineering, 12530 Sichuan University, 24 South Section 1, First Ring Road, 610065 Chengdu, Sichuan Province, China

## Abstract

Microwave heating is widely used because of its advantages of selective heating, internal heating, high efficiency, and easy controllability. With the proposal of efficient production and high-quality products, the advantages of microwave heating such as high efficiency and fast heating speed are becoming more and more apparent. The results of this article have shown that the optimum energy consumption of the microwave source, i.e., the optimum temperature rise curve of the heated material during the microwave heating process, can be obtained by precise control of the microwave source with the switch control circuit. Moreover, for the same material, under the same starting and ending temperatures, identical processing parameters such as heating time and different temperature rise curves could result in different microwave energy consumption. Proper temperature rise curves enable about a 10% reduction in energy consumption during the microwave heating period. Moreover, by controlling the start and stop of the microwave source, ideal temperature rise curves can be obtained—the temperature rise curves not only enable accurate control of the temperature of the heated object to avoid excessive temperature debasing product quality but also can achieve diverse heating purposes such as decreasing energy consumption and improving evaporation speed.

## Graphical abstract

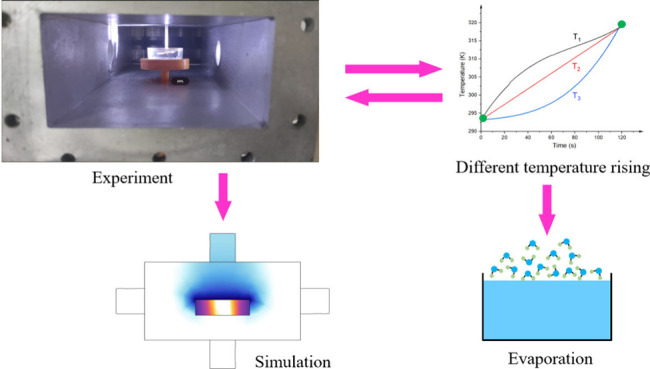

## 1 Introduction

Microwave heating was discovered in the 1950s, and because of its characteristics of fast heating, selective heating, internal heating, and convenient control, this technology has spread quickly throughout the world.^1^ Microwave heating is being used more and more widely along with the process of electrical elements. In pace with the development of research and theories in heating and chemistry, microwave heating is used in various fields such as food engineering, biomass processing, the chemical industry, and so on.^2–15^ Compared with traditional heating and drying methods such as hot air drying, in the microwave drying process, microwaves directly act on the material, and the material is heated from inside. The direction of heat conduction is the same as the direction of moisture migration so that the microwave drying speed is fast, the efficiency is high, and heating time and energy consumption are saved.^1,2^ Nowadays, with the pursuit of environmental protection and “carbon neutrality and carbon standards”, these characteristics are particularly prominent.^16–18^

However, due to factors including magnetron efficiency and the design of the microwave heating cavity, microwave heating still suffers from disadvantages such as low overall efficiency and high energy consumption, making the improvement in its efficiency a persisting important factor.^19–22^

The energy-saving issues in microwave heating and microwave drying processes have been widely studied. Gonźalez-Cavieres studied the vacuum microwave drying process and described the phenomenological behavior of the drying process, noting the optimization and computer simulations method to increase the process efficiency and lower the energy consumption.^4^ Wang et al. indicated the microwave drying process and modified the page I model, which increased the efficiency of microwave heating sewage sludge.^23^ Deleu et al. summarized three distinct generations of microwave technology and looked into the effect of the different generations of microwave technology on the efficiency of the process.^24^ Li et al. proposed the feasibility of applying spouting technology in RFD (radiofrequency drying) and the cooperative working mode to improve the uniformity and efficient of microwave drying.^25^ Zhang et al. offered a comprehensive examination of the heating mechanism, material characteristics, and parameters related to microwave-based material processing, and they expanded the usage scope of microwave heating and analyzed different heated materials such as susceptor material, insulator material, and interface material.^26^ An et al. systematically reviewed the quantification methods and influencing factors of the energy efficiency of microwave drying from different perspectives and proposed future trends in improving the energy efficiency.^27^ Dai et al. used a new structure named EMBH (electromagnetic black holes) to increase the energy conversion efficiency and researched the effect of efficiency with respect to silicon carbide tubes, tube diameter, and lifting and rotating speeds.^28^ Eom et al. combined microwave drying with flush drying and optimized the process to achieve high efficiency sludge drying with low energy consumption.^29^ The research of these scholars has advanced the development of microwave drying technology, providing valuable and innovative insights for engineering applications and the promotion of microwave technology. However, studies on achieving energy savings by controlling the temperature rise curve of microwave heating are still lacking.

This study is based on the fundamental principles of microwave heating,^1,30^ with a focus on the control of microwave sources and energy consumption under different temperature rise curves. Via multiphysics simulation, this study reveals a new correlation among microwave heating, temperature rise curves, and energy consumption, thereby providing reliable insights and design principles for the development of highly efficient, controllable microwave heating technologies and systems.

## 2 Theories

In his experiment, we heated water via microwave and analyzed the evaporated mass and heat dissipation to compare the energy consumption. The calculation model and equations incorporate mathematical descriptions of microwave heating, mass transfer, heat dissipation, and control.

### 2.1 Microwave Heating

This experimental material is water that could be classified as polar molecule material. Water in the microwave is heated mainly by the dielectric polarization’s rotation in the alternating electric field of the microwave. The power dissipation of microwave heating can be calculated by the governing equations as shown below.

#### 2.1.1 Maxwell’s Equation

1 $$\{\begin{array}{l} \nabla \cdot \mathrm{E⃗}=\frac{\rho }{\varepsilon }\\ \nabla \cdot \mathrm{B⃗}=0\\ \nabla \times \mathrm{E⃗}=-\partial \mathrm{B⃗}/\partial t\\ \nabla \times \mathrm{B⃗}=\mu \left(\mathrm{J⃗}+\varepsilon \partial \mathrm{E⃗}/\partial t\right) \end{array}$$

where *E⃗* is the electric field (V/m), ρ is the charge density (C/m^3^), *ε* is the permittivity of the medium (F/m), *B⃗* is the magnetic flux density (V/m), *t* is time (s), μ is the permeability of the medium (H/m), and *J⃗* is the current density (A/m^2^).

#### 2.1.2 Wave Equation

In the progress of microwave heating, microwaves transmit through lossy media, the wave equation is

2 $$\nabla \times \left(\nabla \times \mathrm{E⃗}\right)/\mu_{r}-{\left(\omega /c\right)}^{2}\left(\varepsilon_{r}-j\cdot \sigma /\omega \varepsilon_{0}\right)\mathrm{E⃗}=0$$

where *μ*_*r*_ is the relative permeability (N/A^2^), ω is the angular frequency (rad/s), *c* is the speed of light in vacuum (2.998 × 10^8^ m/s), *ε*_*r*_ is the relative permittivity, σ is the electrical conductivity (S/m), *ε*_0_ is the permittivity of free space (8.85 × 10^–12^ F/m), and *j* is the imaginary unit.

#### 2.1.3 Dissipated Power Density

3 $$Q=\omega \varepsilon_{0}\varepsilon_{eff}^{\prime \prime }{\left|\mathrm{E⃗}\right|}^{2}$$

Here, *ε*_*eff*_^″^ is the relative dielectric loss.

#### 2.1.4 Dielectric Properties of Water

The ability of microwaves to heat a material is mainly evaluated by the complex dielectric constant ε* = ε′ – *j*·ε_*eff*_^″^, where *j* means the imaginary unit. The real part *ε′* (called the “relative dielectric constant”) can be used to estimate the polarization ability of molecules in an electromagnetic field, while ε_*eff*_^″^ is the imaginary part (called the “relative dielectric loss”). According to the Debye model, the complex permittivity of a dielectric material can be estimated as

4 $$\varepsilon^{*}=\varepsilon_{r\infty }+\left(\varepsilon_{rs}-\varepsilon_{r\infty }\right)/\left(1+i\omega \tau \right)$$

where

5 $$\tau =\tau_{0}\exp\left(W_{a}/k_{0}t\right)$$

$$\varepsilon_{rs}=\frac{(3\varepsilon_{r\infty }T+A{\left(\varepsilon_{r\infty }+2\right)}^{2}+\sqrt{{\left(3\varepsilon_{r\infty }T+A{\left(\varepsilon_{r\infty }+2\right)}^{2}\right)}^{2}+72{\varepsilon_{r\infty }}^{2}T^{2}}}{12T}$$ 6

7 $$A=A_{0}\exp\left(-U/k_{0}T\right)$$

*k*_0_ is the Boltzmann constant, τ_0_ is the constant for water, and *W*_*a*_ is the activation energy. This equation gives the relationship between the permittivity and temperature. The electromagnetic and thermal parameters required for the calculation are shown in Table 1.

**Table 1:** Calculation of the Required Electromagnetic and Thermal Parameters

*k*_0_(J/K)	*A*_0_	τ_0_	*U*(J)	*W_a_* (J)
1.38 × 10^–23^	1186.8	6.27 × 10^–15^	–2.88 × 10^–21^	2.96 × 10^–20^

### 2.2 Heat Transfer

In this experiment, water is heated by microwaves, and the temperature of the solution can be calculated with eq 8 ^31^

8 $$\rho C_{p}\frac{\partial T}{\partial t}+\rho C_{p}\mathrm{u⃗}\cdot \nabla T=k\nabla^{2}T+Q$$

where ρ is the density of water (kg/m^3^), *C*_*p*_ is the specific heat capacity of water (J/kg·°C), *T* is the temperature of water (K), *u⃗* is the water’s velocity coming from the intrinsic fluid properties, *k* is the thermal conductivity of water (W/(m·°C)), and *Q* is the microwave heating source and is calculated with eq 3.

### 2.3 Heat Dissipation Calculation

The microwave energy absorbed by water is converted to a temperature rise and is exchanged with the surrounding environment for heat dissipation. The methods of heat dissipation included conduction heat dissipation, evaporation heat dissipation, convection heat dissipation between liquid surfaces and air, and radiation heat dissipation. Due to the static air in the space where deionized water is located and the low water and air temperatures, convective and radiative heat dissipation are ignored in the calculation. This experiment calculates only conduction heat dissipation and evaporation heat dissipation.

#### 2.3.1 Conductive Heat Dissipation

The equation for conducting heat dissipation is

9 $${\mathrm{q⃗}}_{c}=\nabla h$$

where *q⃗*_*c*_ is conduction heat flux (W/m^2^), *h* is the heat transfer coefficient (W/m^2^·K), and *T* is the surface temperature (K).

#### 2.3.2 Evaporation Heat Dissipation

The calculation equations for steady-state evaporation and the heat dissipation of water are

10 $$\{\begin{array}{l} c_{sat}=p_{sat}/R_{g}T\\ q_{e}=Lg_{e}\\ g_{e}=K\left(c_{sat}-c_{V}\right)M_{V} \end{array}$$

where *L* is the latent heat of vaporization (2260 kJ/kg in 373 K), *g*_*e*_ is the evaporation flux (kg), *K* is the evaporation rate (kg/(m^2^·s)), *c*_*sat*_ is the saturated vapor concentration (kg/m^3^), *c*_*v*_ is the vapor concentration (kg/m^3^), *M*_*v*_ is the molar mass of water vapor (kg/mol), *p*_*sat*_ is the saturated vapor pressure (Pa), *R*_*g*_ is the universal gas constant (8.314 Pa·m^3^/(mol·K)), and *T* is the surface temperature (K).

### 2.4 Control

On the basis of the comprehensive experimental conditions, experimental accuracy, and reliability of the control system, traditional switch control was adopted in the experiment. We controlled the starting and stopping of the solid-state source using the heating time and temperature of the water as dual feedback and judging conditions. If the preset heating time was reached or the measured temperature was higher than the preset temperature, then the solid-state source was turned off. When the measured temperature was equal to or less than the preset temperature, the solid-state source was turned on. The control flowchart is shown in Figure 1.

**Figure 1:**
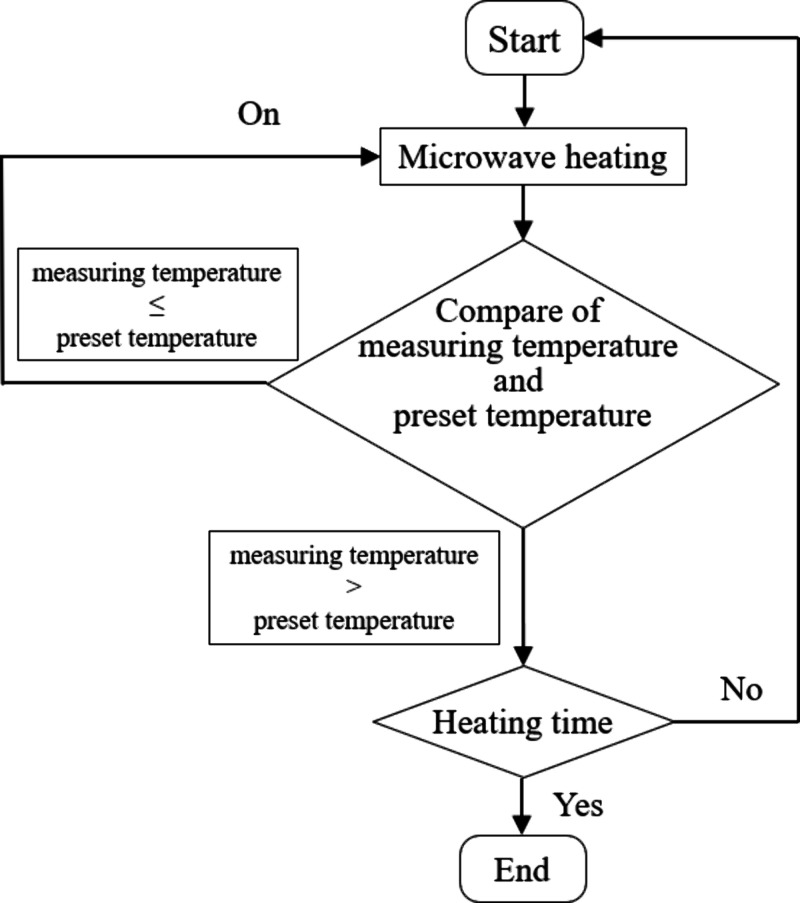
Control flowchart.

## 3 Experiment

### 3.1 Design of Experiment

To ensure the accuracy of input power, a solid-state source was used as the microwave source in the experiment.^32^ In this experiment shown in Figure 2, solid-state source output microwaves of 2450 MHz^1^ were transmitted on a coaxial cable to the waveguide converter. We used the microwaves transmitted through a circulator and a no. 1 dual directional coupler and then into the waveguide where the material (deionized water) is heated. The microwaves continued transmitting to the no. 2 dual directional coupler and were ultimately absorbed by the last water load.

**Figure 2:**
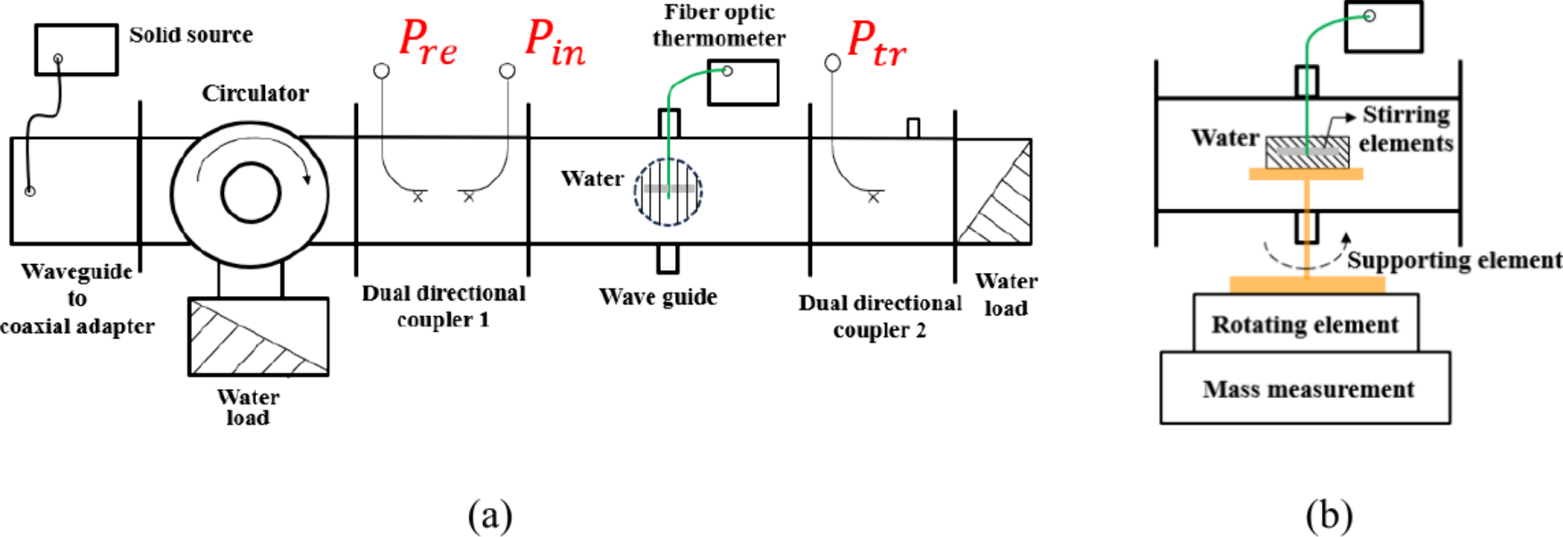
Simplified 2D drawing of the experimental system: (a) top view and (b) main view of the cavity.

In this experiment, two dual directional couplers were used to respectively measure the input power (*P*_*in*_), reflected power (*P*_*re*_), and transmission power (*P*_*tr*_). The absorbed power (*P*_*absorb*_) of the heated material can be calculated by eq 11:

11 $$P_{absorb}=P_{in}-P_{re}-P_{tr}$$

In order to ensure the uniformity of the temperature distribution of the heated object, a rotating table was placed under the support frame of the heated material. The rotating table drove the support frame, and the heated material was rotated in pace with the support frame. A stirring device was placed in the heated material to further improve the overall temperature uniformity of the heated object.

To ensure that the mass measurement is accurate with minimal latency, this part of the experimental system was composed of rotation, stirring, and electronic balance (weighing platform). The deionized water was placed on a support frame, under which lay a rotary device and an electronic balance to weigh the mass change. When the rotary device operated at a stable and preset speed, it drove the internal deionized water to rotate. Inside the deionized water, the stirrer manufactured by PTFE remained stationary so that it maintained a fixed relative position with respect to the waveguide system, while the water was stirred by the PTFE-manufactured stirrer. The relative motion between the stationary stirrer and rotating deionized water caused water to be stirred during the heating process, thereby allowing the deionized water to be heated uniformly during the heating process. The electronic balance was placed under the deionized water, support frame, and rotary table, allowing real-time measurement of mass changes and calculation of the evaporation mass based on the variations of the electronic balance display reading.

**3:**
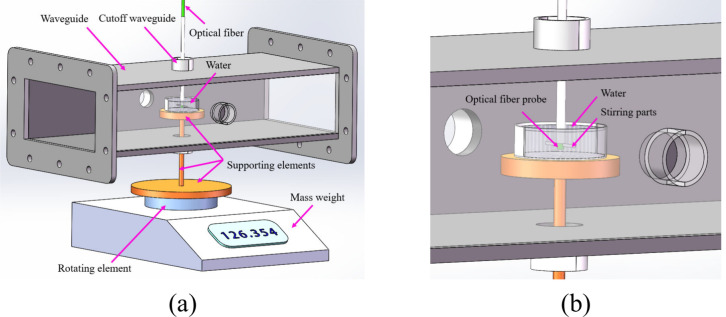
Schematic drawing of experiment cavity: (a) mass measurement, rotating, and temperature probe and (b) inner experiment cavity and water.

### 3.2 Experimental Setup

The experimental setup is shown in Figures 4 to 6.

**Figure 4:**
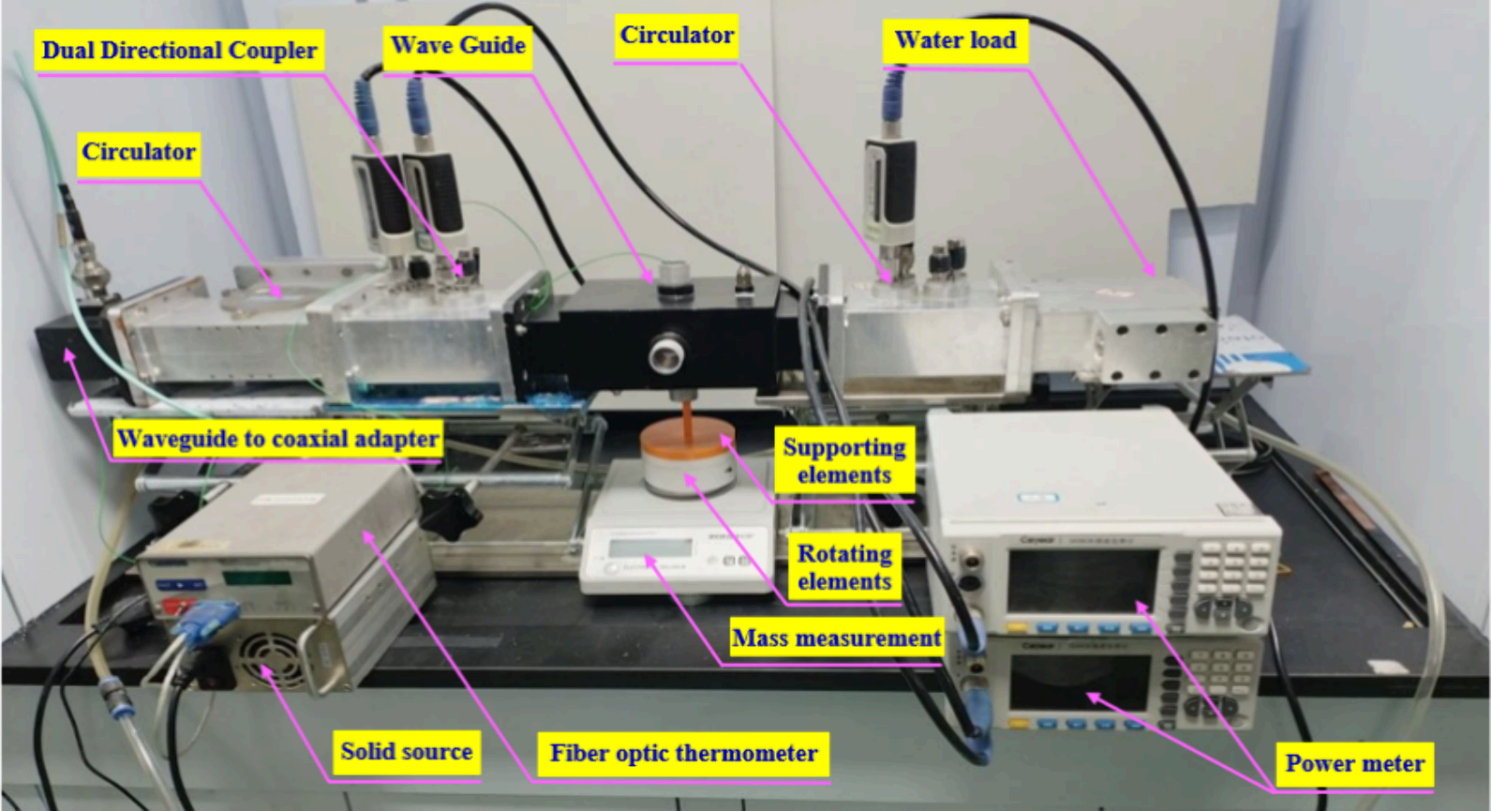
Experimental setup.

**5:**
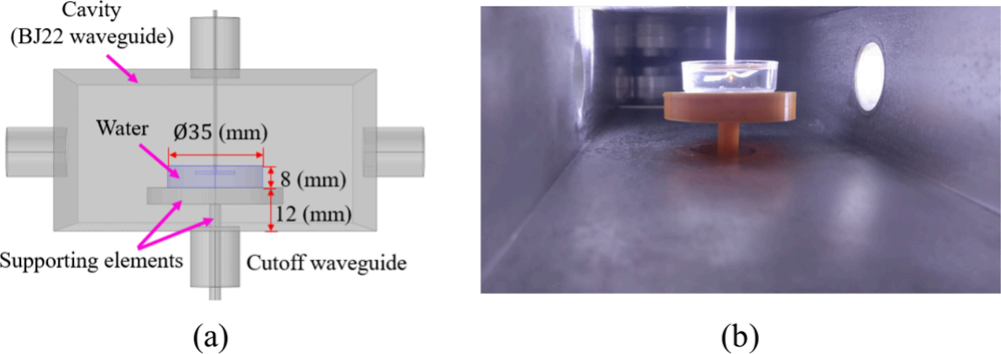
Schematic drawing of heated material: (a) model of simulation and (b) inside the waveguide.

**Figure 6:**
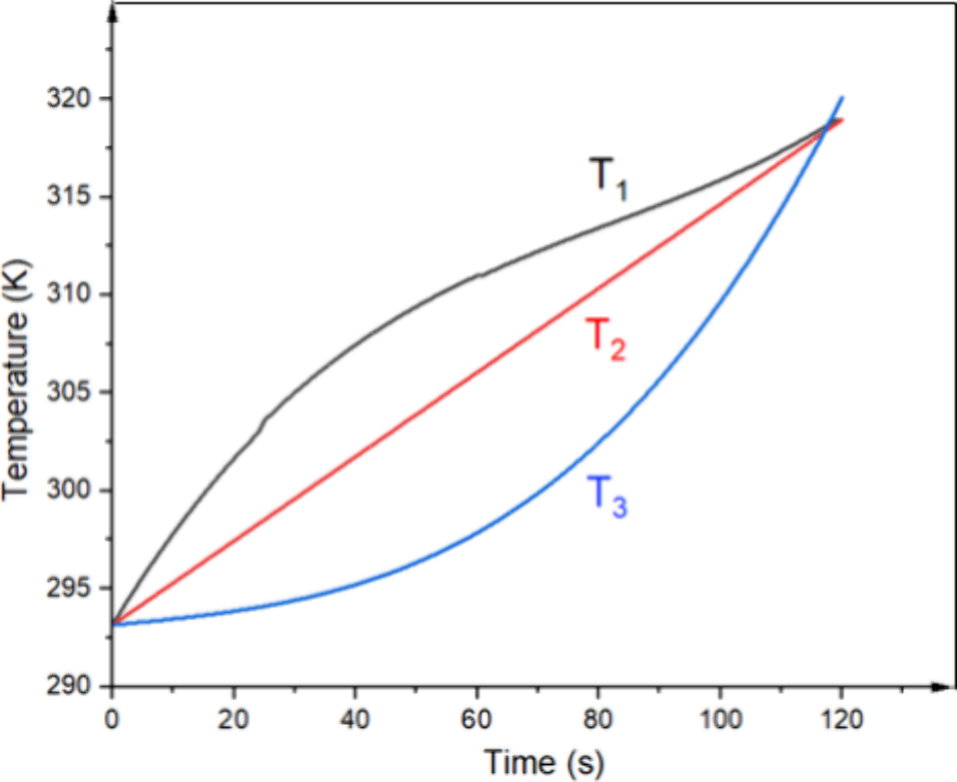
Preset temperature rise curves.

In this experiment, we set up three different temperature rise curves and used the ON/OFF of the microwave source through temperature feedback of the heated material to achieve preset temperature rise curves. The equations for the three preset temperature rise curves are shown as

12 $$T_{1}=\left(0.0000379\cdot t^{3}-0.0094\cdot t^{2}+1.006\cdot t\right)\cdot 0.51+293.15$$

13 $$T_{2}=0.215\cdot t+293.15$$

14 $$T_{3}=\left(0.0000135\cdot t^{3}+0.03\cdot t\right)+293.15$$

The three preset temperature rise curves are shown in Figure 6.

### 3.3 Experiment Results

According to the image taken by the infrared thermal imager,^33^ it could be seen that the temperature distribution of deionized water was relatively uniform under the action of rotation and stirring, with a surface temperature difference of less than 1 °C. The temperature distribution is shown in Figure 7.

**Figure 7:**
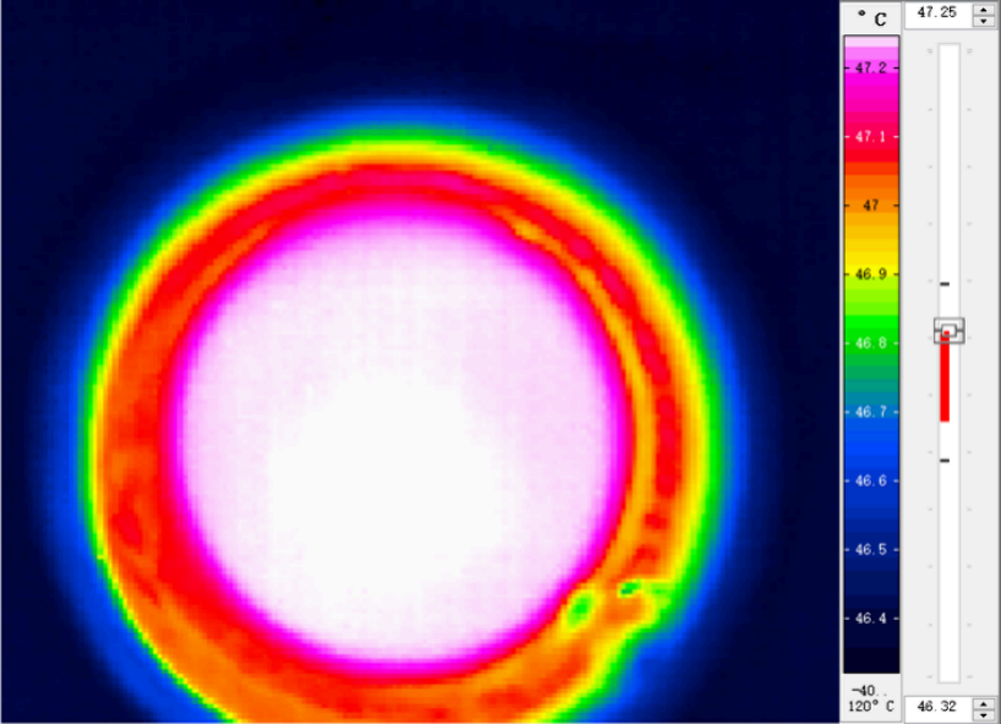
Surface temperature of water.

According to the results of the fiber optic thermometer, the temperature rise curves are shown in Figure 8 (raw data can be seen in the Supporting Information).

**Figure 8:**
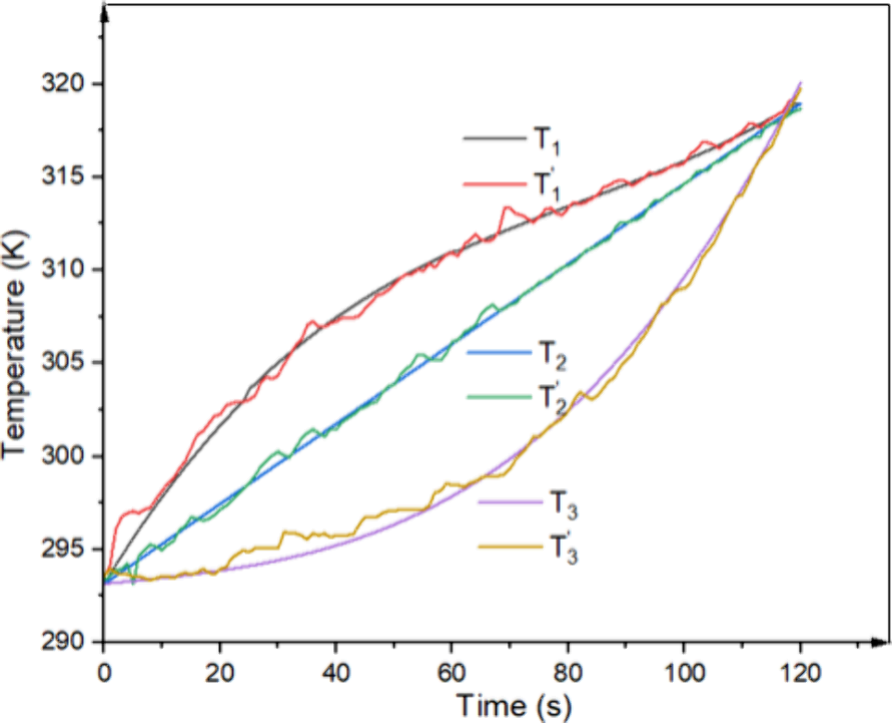
Measured temperature and preset temperature curves of water.

To perform the accuracy validation in order to calculate the degree of fitness between preset temperature curves and the measured temperature rise curve, we calculated their Pearson correlations by Origin.

According to the results of power meters and the electronic balance, the evaporation mass and absorbed power are shown in Figure 9 and Table 3 (raw data can be seen in the Supporting Information).

**Table 2:** Pearson Correlations of Temperature Curves between the Experiment and Preset Values

Temperature curves	Pearson Correlations
T_1_ and T_1_′	0.997
T_2_ and T_2_′	0.999
T_3_ and T_3_′	0.998

**Figure 9:**
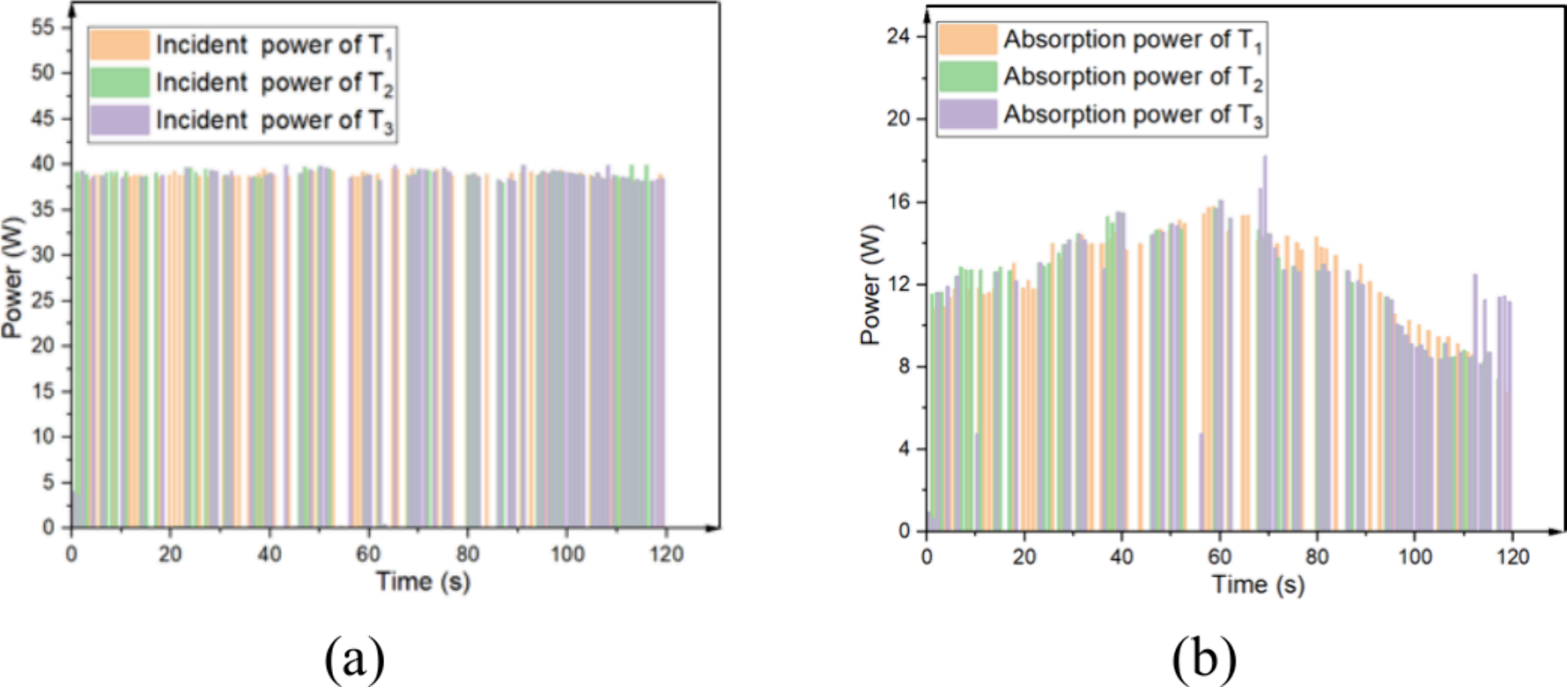
Comparison of power: (a) incident power and (b) absorption power.

**Table 3:** Mass of Evaporation and Absorption Energy

Heating method	Evaporation mass (g)	Absorbing energy (J)
T_1_	0.073	849.69
T_2_	0.065	758.45
T_3_	0.060	671.66

## 4 Results and Discussion

### 4.1 Results

In the simulation of the COMSOL model, we use the control strategy of the temperature heating curves. As mentioned in section 2.4 and Figure 1, the solid-state source is controlled by temperature and recorded by the power meter as shown in Figure 4. When the solid-state source is On, the power meter displays and records the output power; when it is Off, the power meter shows an output power of 0. The power meter records could be used not only to calculate the energy but also to monitor the solid-state source’s working state. Based on the recorded output power, operating time, and stop time from the power meter, simulations and calculations are performed by using COMSOL. Using multiphysics simulation software COMSOL,^34–36^ we calculated the convection heat dissipation values and evaporation heat dissipation values of three heating methods. The results are shown in Tables 4 and 5.

**Table 4:** Calculation Value of Conduction and Evaporation Heat Dissipation from COMSOL

Heating method	Convection heat dissipation (J)	Evaporative heat dissipation (J)	Total heat exchange value (J)
T_1_	78.57	215.27	293.84
T_2_	50.96	150.03	200.99
T_3_	28.03	104.84	132.87

**Table 5:** Evaporative Heat Dissipation COMSOL Calculation Values

Heating methods	Heating sensible heat (J)
T1	555.85
T2	557.46
T3	538.79

According to eq 11, we used the calculated heat dissipation from the measured microwave energy absorbed by deionized water to obtain the heating sensible heat of water. The heating sensible heat of the three heating methods is the same,^37^ so it can be considered that the calculation is accurate. The calculation results are shown in Table 5.

On the basis of the T_2_ value, the errors in T_1_ and T_3_ are −0.3 and −3.4%, respectively.

In the COMSOL simulation, the temperature distribution images of the cross-section at the end of the heating process are shown in Figure 10.

**Figure 10:**
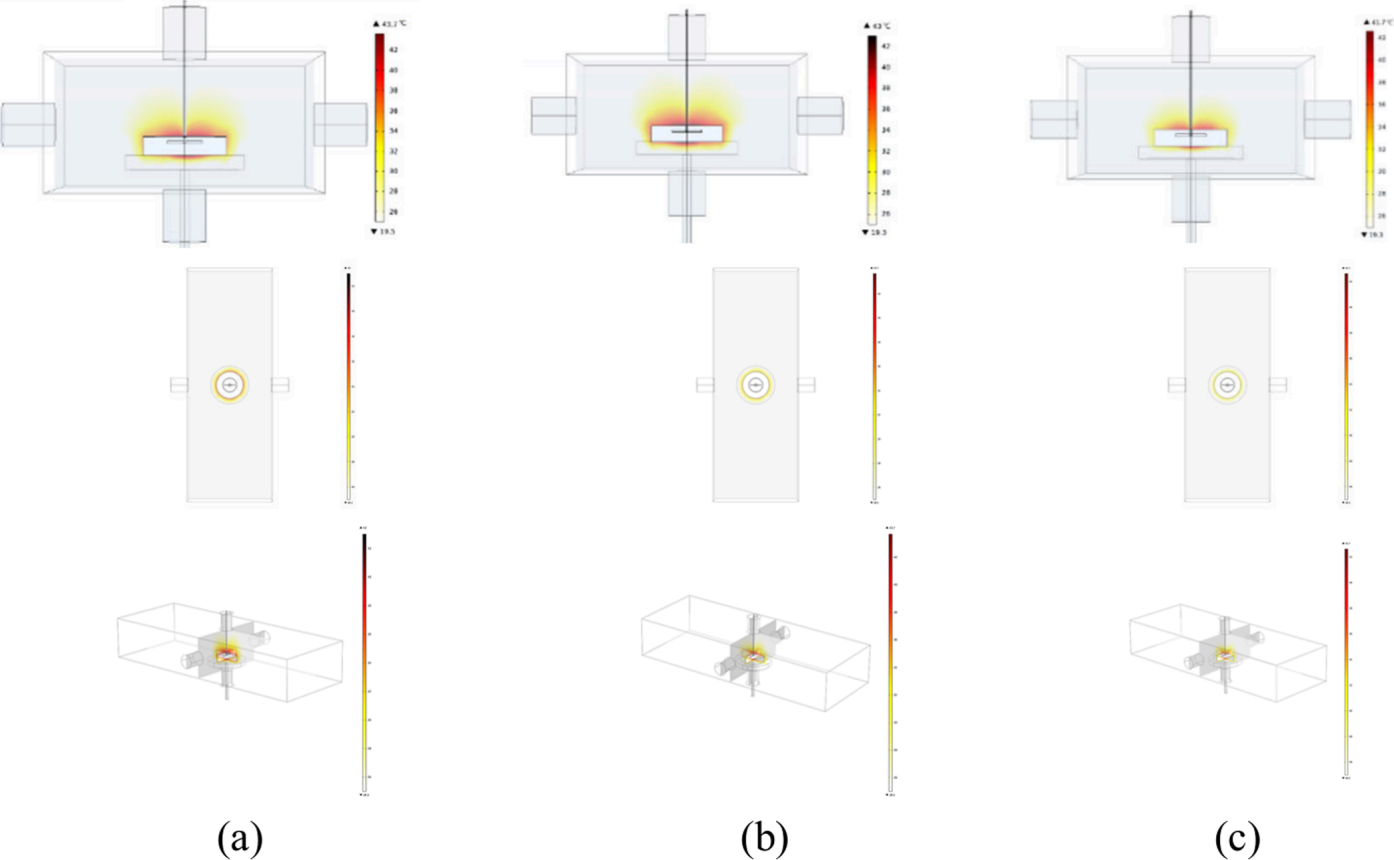
Temperature distribution of side view, top view, and oblique view from COMSOL: (a) heating method of T_1_; (b) heating method of T_2_; and (c) heating method of T_3_.

According to the calculation results, it can be seen that in temperature curve 1 there is a significant amount of heat dissipation.

For temperature rise curve T_1_, due to being in a high-temperature area for a longer period of time, the evaporation rate is high and there is more heat dissipation to the outside, and under the heating condition of temperature rise curve T_1_, the vapor concentration on the liquid surface is the highest, which also affects the heat dissipation of evaporation. A similar process also occurs during the heating of temperature rise curves T_2_ and T_3_. Further analysis indicates that when materials are heated by different methods their sensible heat is the same given the same starting and ending temperatures, which can be confirmed in Table 4. However, variations in the temperature rise curves lead to differences in heat exchange and heat dissipation with the surroundings, consequently resulting in different energy consumption levels.

### 4.2 Discussion

According to the data in Tables 3 to 5, we compared the data of T_1_ and T_3_ based on the heating method of the T_2_ temperature rise curve. The results are summarized in Table 6.

**Table 6:** Comparative Data on Evaporation Quality, Energy Consumption, and Heat Exchange

Heating methods	Evaporation quality change rate	Energy consumption and rate of change	Heat exchange and rate of change
T_1_	10.61%	12.02%	43.49%
T_3_	–9.09%	–11.44%	–30.12%

By comparing the data in Table 2, it can be seen that (1) when using the T_1_ temperature rise curve for heating, the evaporation mass increases by 25.86% and the energy consumption increases by 28.61%; (2) when using the T_2_ temperature rise curve for heating, the evaporation mass increases by 12.07% and the energy consumption increases by 14.80%; and (3) when using the T_3_ temperature rise curve for heating, the energy-saving effect is the most significant.

## 5 Conclusions

This experiment aims to study the energy consumption of a liquid heated by microwaves. A temperature-based feedback control system is used to regulate the activation and deactivation of the microwave source, enabling the generation of different temperature rise curves for the analysis of the energy consumption in the heating process. According to the experimental results, the following conclusions can be drawn:

(1) The temperature rise curve of the heated object during microwave heating can be easily and accurately controlled through the temperature-based feedback control system.
(2) When the starting temperature and final temperature are the same, different temperature rise curves result in different evaporation rates and different energy consumption of the system.
(3) By controlling the temperature rise curves of the heated object, different temperature rise curves can achieve different production goals, such as increasing the evaporation mass, reducing the unit evaporation energy consumption, and decreasing the external heat exchange.

However, this experimental cavity is a single-mode cavity modified with a BJ22 waveguide. When the cavity is changed to a multimode cavity, the liquid mass also increases, and when the microwave source is changed to a high-power microwave source, it is worth further discussion of whether the same effect still exists.

## Supplementary Material



## Notes

Raw experiment data, including temperature and power data (XLSX)
